# Analysis of organ-enriched microRNAs in plasma as an approach to development of Universal Screening Test: feasibility study

**DOI:** 10.1186/1479-5876-11-304

**Published:** 2013-12-11

**Authors:** Kira S Sheinerman, Vladimir G Tsivinsky, Samuil R Umansky

**Affiliations:** 1 DiamiR, LLC, 11 Deer Park Drive, Suite 102G, Monmouth Junction, NJ 08852, USA

**Keywords:** Biomarker, miRNA, Circulating miRNA, miRNA pairs, Cancer, Inflammation, Pulmonary system, Gastrointestinal system, Plasma, Screening

## Abstract

**Background:**

Early disease detection with a minimally invasive screening test will significantly increase effectiveness and decrease the cost of treatment. Here we propose a framework of a novel approach – Universal Screening Test (UST) for the detection of pathological processes in a particular organ system, organ, or tissue by RT-qPCR analysis of circulating cell-free miRNAs in plasma. As the first step towards assessing the feasibility of this concept, the present study was designed to analyze whether the same microRNAs (miRNAs) can detect various diseases of a particular organ system.

**Methods:**

RNA was extracted from plasma using Trizol treatment and silica binding. Levels of miRNAs were measured by single target RT-qPCR. The following innovations have been tested and proven effective: (i) the use of organ system/organ/tissue-enriched miRNAs; (ii) the use of miRNAs associated with broad disease categories, such as cancer and inflammation, in combination with the organ-enriched miRNAs; and (iii) the use of “miRNA pairs” for selecting miRNA combinations with the highest sensitivity and specificity.

**Results:**

Here we report biomarker miRNA pairs effectively differentiating (i) patients with pulmonary system diseases (asthma, pneumonia and non-small cell lung cancer) and gastrointestinal (GI) system diseases (Crohn’s disease, stages I/II esophageal, gastric and colon cancers) from controls, each with 95% accuracy; (ii) patients with a pathology of the pulmonary system from patients with a pathology of the GI system with 94% accuracy; and (iii) cancer patients (stages I/II esophageal, gastric, colon cancers, or non-small cell lung cancer) from patients with inflammatory diseases (asthma, pneumonia, or Crohn’s disease) with 93%-95% accuracy.

**Conclusions:**

The results obtained in the present study, along with the data reported by us and others previously, are encouraging and lay the ground for further investigation of the described approach for UST development.

## Background

It is well accepted that treatment of a disease is more effective if an underlying pathology is diagnosed early. For some diseases, early diagnosis, preferably made prior to manifestation of clinical symptoms, is of critical importance, because of pathology’s progression to an advanced, sometimes irreversible, stage. For example, one of the major problems facing drug development and treatment of Alzheimer’s and other neurodegenerative diseases is their late clinical manifestation: due to high compensatory potential of the brain the diseases are usually diagnosed when many neurons are already dead [1]. As a consequence, current treatments aim to delay or prevent worsening of the pathology, rather than to reverse the course of the disease. Another example is cancer, where treatment of an invasive and metastatic disease is much more difficult than treatment of a primary tumor [2,3].

A major advantage of many existing screening tests is their disease-specificity. However, there are hundreds of human diseases, and it is difficult to envisage the development of a specific screening test for each pathology. Moreover, even if specific tests for early detection of all human diseases were developed, application of a large number of tests for screening of broad populations will likely be impractical due to limited accuracy and high costs. For example, if a screening test for a disease with prevalence 1:10,000 is 100% sensitive and 99% specific (i.e. highly accurate) and 1 million people are screened, 100 cases would be detected correctly but about 10,000 people would receive false positive results. Concurrent application of a large number of even highly accurate tests will therefore result in a large number of false positive predictions. These considerations provide a motivation for the work reported herein.

In this paper we describe a framework for the development of a novel approach to screening, which specifically detects the presence of a pathological process in a particular organ system, organ, tissue, and/or cell type without diagnosing a particular disease. Testing with UST could be performed periodically to detect presence of pathology in a particular organ or organ system of a person early, preferably at clinically asymptomatic stage. Such testing would then be followed by more expensive and possibly invasive diagnostic tests for a more specific diagnosis.

UST should satisfy a number of criteria. First, because the test will address a large population, it should be minimally invasive or noninvasive. Secondly, UST should not be based on inducing factors or pathogenesis of diseases, since these are specific to a particular disease and cannot be accounted for in a single test. Thirdly, UST should be cost efficient - utilize a limited number of biomarker types, which can be analyzed by the same technique.

Biomarkers suitable for UST should possess the following properties: (i) cell/tissue/organ-specificity or significant enrichment; (ii) ability to appear in extracellular space and to pass various barriers within the body; (iii) presence and reliable detectability in bodily fluids that can be obtained by minimally invasive methods; and (iv) stability. Certain tests currently used in clinical practice employ biomarkers with many characteristics of potential UST biomarkers, e.g. measurement of liver enzymes, or of cardiomyocyte-specific proteins circulating in the bloodstream for the detection of liver and heart pathologies, respectively.

We hypothesized that miRNAs are suitable candidates to serve as UST biomarkers. miRNAs are stable small molecules (~22 nt), which can cross blood–brain, kidney, and placenta barriers and appear in the extracellular space and bodily fluids [4,5]. Some of the methods used for analysis of miRNAs, such as real-time reverse transcription quantitative polymerase chain reaction (RT-qPCR), are specific and sensitive [6]. Most importantly, miRNAs constitute a large class of diverse molecules with some miRNAs enriched in particular organ systems, organs, tissues and cells [7-11].

Although mechanisms of miRNA appearance in the extracellular space and subsequently in the bodily fluids are not well understood, a role of cell death, blebbing, exosome secretion and of other forms of active secretion has been demonstrated [12,13]. Recently we have shown that Mild Cognitive Impairment (MCI), a heterogeneous syndrome characteristic of early stages of various neurodegenerative diseases, including Alzheimer’s and Parkinson’s diseases, can be detected by analysis of brain-enriched miRNAs in plasma [14].

Two novel elements were seen as essential: (i) identification of potential biomarkers among organ-enriched miRNAs, whose changes in plasma concentrations are likely to be caused by a pathology of the respective organ, and whose expression in blood cells is usually low, the latter preventing contamination of these circulating miRNAs due to hemolysis; and (ii) analysis of miRNA ratios to identify pairs providing the best sensitivity and specificity.

In the present feasibility study we demonstrate that the same approach can be used to detect diseases of two other organ systems, GI and pulmonary systems, and to differentiate the gastrointestinal pathologies from the pathologies of the pulmonary system. While the primary disease target of the presented approach is detection of cancer, to demonstrate that same approach can be used to detect other pathologies, several inflammatory diseases are included.

## Methods

### Plasma samples

K_2_EDTA (Ethylenediaminetetraacetic acid dipotassium) plasma samples from 70 patients and 30 controls were obtained from a commercial vendor Bioreclamation (Westbury, NY). The samples were collected in compliance with Health Insurance Portability and Accountability Act (HIPAA), and a written consent was obtained from each subject. DiamiR employees had no access to private patient information. Blood samples were centrifuged at 2,100xg for 15 min at 4°C, aliquoted and frozen at -20°C within 2 hours from blood collection, then transferred to -80°C, and stored and shipped at -80°C. Demographic characteristics of the study groups are shown in Additional file 1: Table S1.

### Plasma RNA extraction and RT-qPCR miRNA analysis

miRNA isolation and RT-qPCR analysis in all experiments were performed by Asuragen Inc. according to the following protocol. RNA was extracted from 200 μl aliquots using Asuragen’s proprietary protocol, which is based on Trizol treatment and silica (Ambiom Glass Fiber Microcolumn) binding. Single target RT-qPCR was performed using the TaqMan® Reverse Transcription Kit (Applied Biosystems (AB) Catalog number 4366596) and miRNA-specific stem-loop primers (see Additional file 1: Table S2 for AB Catalog IDs). RT step was performed in triplicate and 2 μl plasma equivalents were present in final PCR performed in ABI 7900HT instrument. Cq were determined with SDS v2.3 software. Placental RNA was used as a “positive control” and No-Template controls were run as a “negative control” with every run. The concentrations of the 18 miRNAs (see Additional file 1: Table S2) were determined in the plasma samples of patients and controls as described in Results.

### Statistical methods

In addition to biological factors, such as levels of miRNA expression and secretion and blood supply to different organs, miRNA yield from plasma and the level of RT-PCR inhibitors in the blood of a given subject affect miRNA measurements [15]. Therefore, data normalization is an issue of critical importance. Herein, we employ the “biomarker miRNA pair” approach used in our previous study [14] and by others [16,17]: normalization is based on an experimental search for miRNA pairs, which most effectively differentiate two populations, e.g. pathology versus control; to this end, ratios of levels of all possible miRNA pairs from the same sample are calculated (2^-ΔCq^) and the most promising pairs are selected. The advantage of this approach is that in certain cases miRNAs, whose concentrations are changed due to a pathology in opposite directions, can form a pair effectively differentiating investigated populations.

All statistical calculations were performed with the use of custom software developed at DiamiR [14]. Mann–Whitney *U*-tests were used to evaluate significance of differentiation of any two patient groups by various biomarker miRNA pairs. Bonferroni correction was applied for estimating significant P-values. In experiments on differentiation of GI and pulmonary system pathologies from control 8 miRNAs were tested, thus P-value < 0.0018 (calculated as 0.05/28; 28 here indicates the total number of miRNA pairs examined) was considered significant. In experiments on differentiation of diseases of GI system from the diseases of pulmonary system and on differentiation of cancers and inflammatory diseases 18 miRNAs were tested, thus P-value < 0.0003 (calculated as 0.05/153; 153 here indicates the total number of miRNA pairs examined) was considered significant.

A standard formula for a case–control study [18] provides an estimate of the sample size required to produce a certain statistical power (usually between 0.80 and 0.90). For the P-values given in the paragraph above, and the ratio of difference between averages for comparison groups to standard deviation of 2, the sample size calculated according to ref. 18 to provide for power = 0.95 is 42 samples for the detection of GI and pulmonary system pathologies, 52 samples for the differentiation of pathologies of two organ systems, and 52 samples for the differentiation of cancers from inflammatory diseases. In the present study, a higher number of samples (50 samples for the detection of pulmonary system pathologies, and 70 samples in all other experiments) was used to assure high power of the statistical test.

Receiver-Operating Characteristic (ROC) curves were constructed and the area under ROC curves (AUC) was calculated to evaluate sensitivity and specificity of various miRNA pairs. Sensitivity and specificity are reported for the cutoff points on the ROC curves, which provide the best overall accuracy.

## Results

### Selection of miRNAs

Potential organ-enriched biomarker miRNAs were selected according to the following two-step process. First, miRNAs reported in the literature [7-11] to be enriched (when compared with other organ systems) in normal tissues and organs of pulmonary and GI systems were selected. Second, we examined published reports describing analyses of circulating cell-free miRNAs in plasma and serum [19-22] and selected from the pool of miRNAs chosen in the first step only miRNAs reported to be detectable in the bloodstream. The second step in the selection process is critical, since levels of miRNA expression in various tissues generally do not correlate with their concentrations in plasma or serum [23,24], and the plasma levels of tissue-enriched miRNAs are usually significantly lower than the levels of ubiquitous miRNAs. The miRNAs selected for testing are listed in Additional file 1: Table S2.

miRNAs shown to be involved in carcinogenesis and inflammation [25-29] were also included in the analysis (see Additional file 1: Table S2), so as to investigate a possibility of differentiating these most common types of pathological processes.

### Detection of GI system pathologies

**Figure 1:**
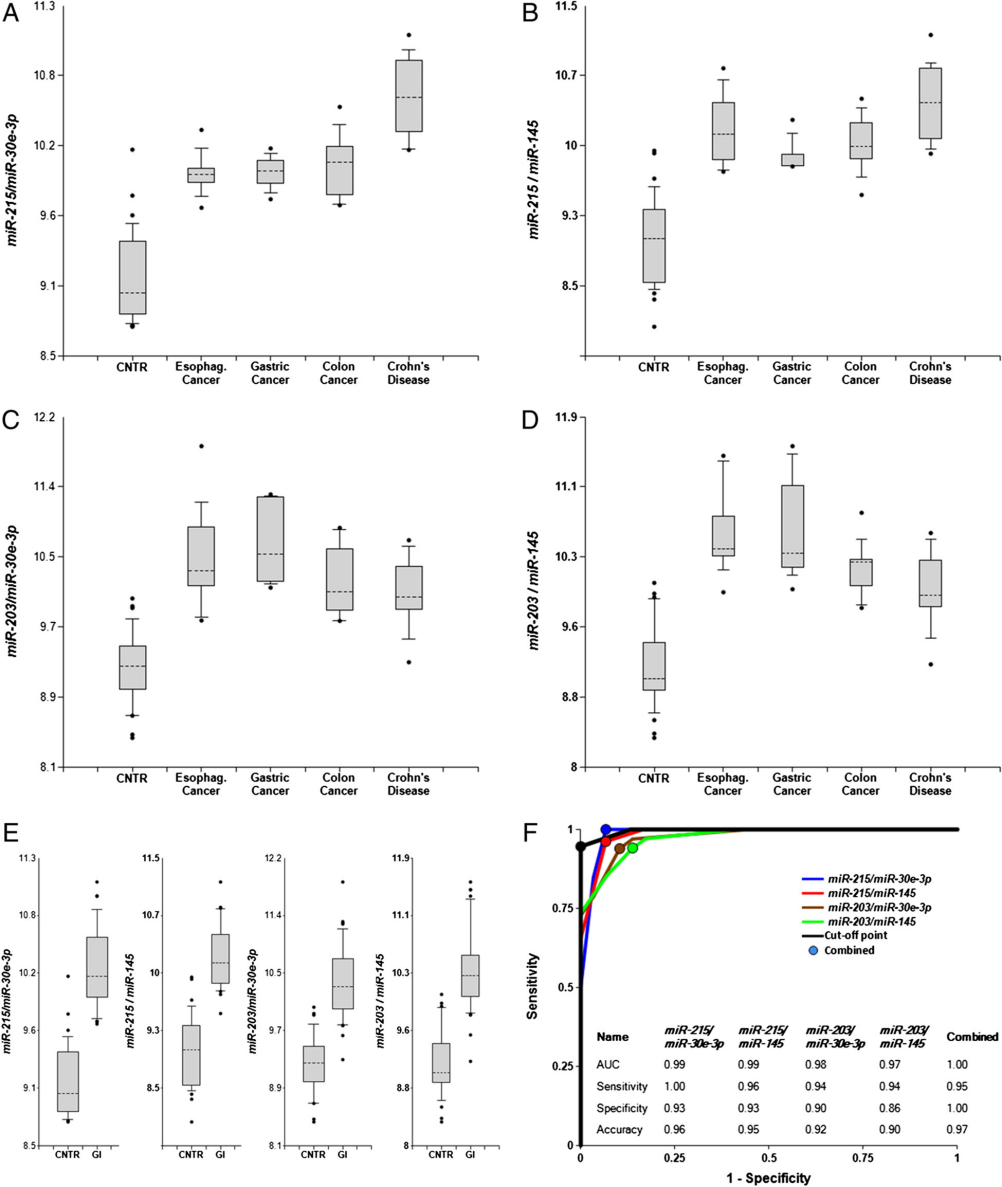
**Differentiation of GI pathologies from controls by miRNA biomarker pairs.** The concentrations of miRNAs in plasma samples of patients with four GI pathologies, and healthy donors were measured by RT-qPCR and the ratios of levels of various miRNAs were calculated as 2^-ΔCq^ x 100. **A**-**E** – box-plots. Here and in other figures with box and whisker plots the results are presented in the log_10_ scale. The upper and lower limits of the boxes and the lines inside the boxes indicate the 75th and 25th percentiles and the median, respectively. The upper and lower horizontal bars denote the 90th and 10th percentiles, respectively. The points indicate assay values located outside of 80% data. **A**-**D** – individual pathologies (10 patients in each group) against controls (30 subjects); **E** – combined GI pathologies (40 patients total) against controls (30 subjects). **F** –ROC curves of differentiation between patients with four GI pathologies and controls obtained with different biomarker pairs. The areas under the ROC curves (AUC) are reported. Sensitivity, specificity and accuracy for each miRNA pair are calculated for the “cutoff” point (indicated as a dot on each plot) – the value of the ratio where the accuracy of predictions is the highest (see Methods, ref. 14).

Plasma samples were obtained from 40 patients with pathologies of the GI system (esophageal, gastric and colorectal cancers, stages I and II, and Crohn’s disease, 10 patients in each group) and 30 healthy controls (see Additional file 1: Table S1). The levels of *miR-145*, *miR-148a*, *miR-192*, *miR-194*, *miR-203*, and *miR-215*, enriched in organs of the GI system, as well as of ubiquitous *miR-30e-3p* and *miR-181a* (see Additional file 1: Table S2) were determined by the single target TaqMan® miRNA RT-qPCR assay. The ratios of all possible miRNA pairs were calculated (2^-ΔCq^) and the ability of each pair to differentiate patients with a GI pathology from controls was analyzed (see Methods). Using sums of two or more miRNAs in the numerator or denominator of a ratio was tested and found not to improve accuracy. Thus, only biomarker miRNA pairs and their combinations are presented. Figure 1 and Additional file 1: Figure S1 demonstrate that certain miRNA pairs effectively differentiate patients with the GI pathologies from controls – both when all pathologies are grouped together, and when analyzed separately. These effective pairs include GI system-enriched miRNAs and ubiquitous *miR-30e-3p*, and GI system-enriched miRNAs paired with each other. The ROC curves for selected miRNA pairs and their combination are presented in Figure 1, panel F. The areas under the ROC curve (AUC) for *miR-215/miR-30e-3p*, *miR-215/miR-145* and *miR-203/miR-145* are 0.97-0.99, and overall accuracy is 90%-96%. P-values presented in Table 1 are 10^6^-10^7^ times lower than P = 0.0018 considered significant with Bonferroni correction (see Methods). Additional file 1: Figure S1, panel H reports additional miRNA pairs able to effectively differentiate patients with the GI pathologies from controls.

**Table 1:** Differentiation of various groups of patients from each other, and from healthy controls with the identified miRNA pairs

**Groups of subjects compared**	**Biomarker miRNA pairs**	**P-value**
GI system diseases versus controls	*miR-215/miR-30e-3p*	2.60E-09
	*miR-215/miR-145*	6.30E-10
	*miR-203/miR-30e-3p*	1.80E-09
	*miR-203/miR-145*	1.20E-10
Pulmonary system diseases versus controls	*miR-486-5p/miR-409-3p*	4.10E-09
	*miR-486-5p/miR-146-5p*	1.70E-10
	*miR-34b/miR-409-3p*	2.20E-06
	*miR-34b/miR-146-5p*	7.40E-06
GI system diseases versus Pulmonary system diseases	*miR-145/miR-155*	7.60E-11
	*miR-486-5p/miR-155*	7.90E-11
	*miR-145/miR-30e-3p*	7.80E-06
	*miR-192/miR-31*	9.80E-06
Cancers versus Inflammatory diseases	*miR-126/miR-30e-3p*	9.90E-08
	*miR-31/miR-155*	8.30E-09
	*miR-146b-5p/miR-30e-3p*	1.77E-09
	*miR-146b-5p/miR-155*	5.86E-10

All P-values given in the table are highly statistically significant. The threshold for statistical significance, as calculated with the Bonferroni correction, is 1.8E-03 for “GI system diseases versus controls” and for “Pulmonary system diseases versus controls”; and 3.0E-04 for “GI system diseases versus Pulmonary system diseases” and for “Cancers versus Inflammatory diseases” (see Methods).

### Detection of pulmonary system pathologies

**Figure 2:**
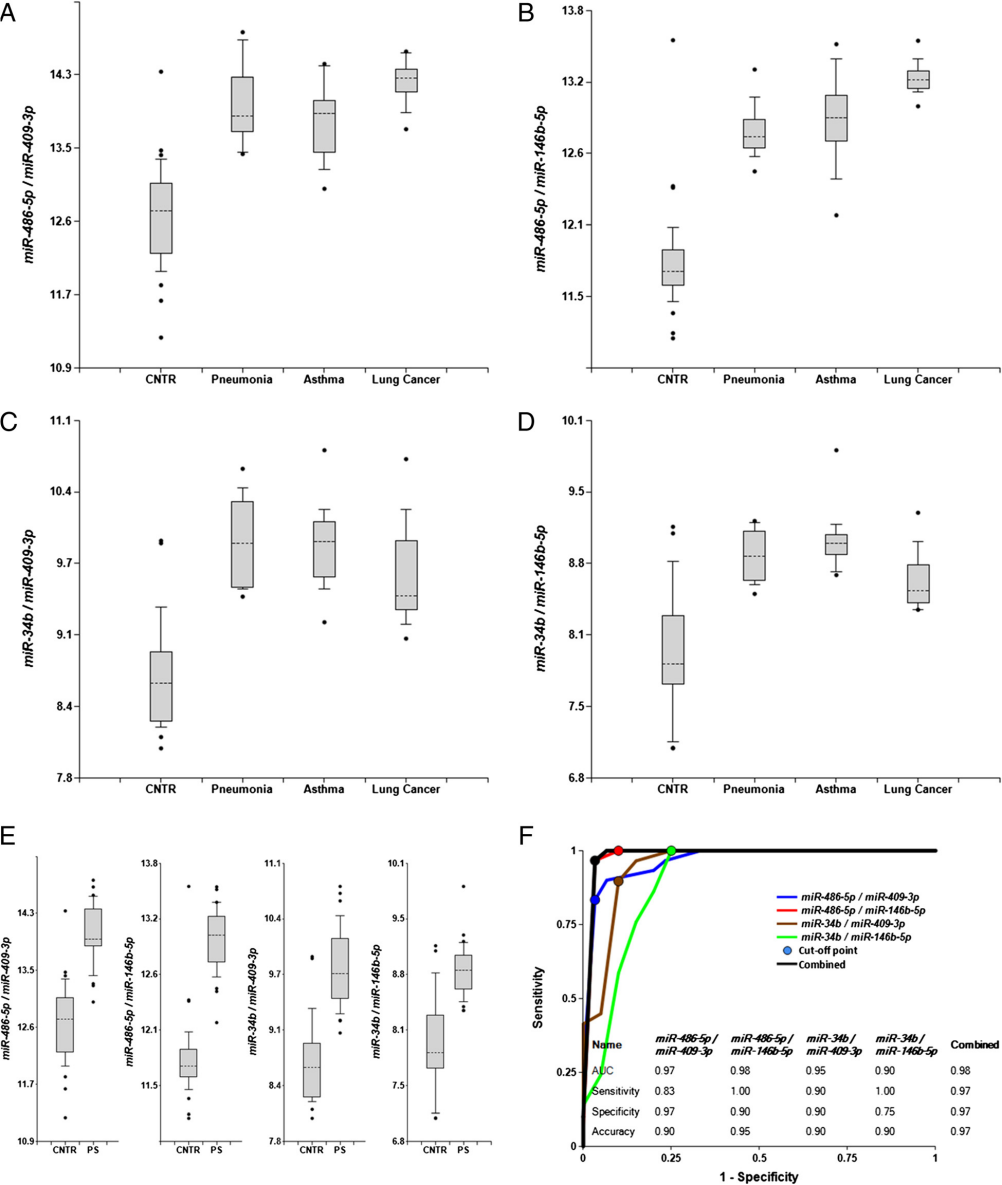
**Differentiation of Pulmonary system (PS) pathologies from controls by miRNA biomarker pairs.** The concentrations of miRNAs in plasma samples from patients with three PS pathologies, and from healthy donors were measured by RT-qPCR and the ratios of various miRNAs were calculated as 2^-ΔCq^ x 100. **A**-**E** – box-plots; **A**-**D** – individual pathologies (10 patients in each group) against controls (20 subjects); **E** – combined PS pathologies (30 patients total) against controls (20 subjects). **F** –ROC curves of differentiation between patients with three pathologies and controls obtained with different biomarker pairs. The statistical analysis is performed as in Figure 1.

Plasma samples were obtained from 30 patients with pathologies of the pulmonary system (asthma, pneumonia and non-small cell lung cancer (NSCLC), stages I - IV, 10 patients in each group) and 20 healthy controls (see Additional file 1: Table S1). Among 20 controls, 10 were smokers and 10 were non-smokers. A smoking status appeared not to affect detection of pulmonary pathologies, and we used the combined control group for the study. The levels of *miR-34b*, *miR-142-5p*, *miR-146b-5p*, *miR-155*, *miR-223*, and *miR-486-*5p, enriched in the pulmonary system, as well as of ubiquitous *miR-409-3p* and of *miR-192*, which is enriched in the GI system, but is also broadly expressed in other organs including pulmonary system (see Additional file 1: Table S2), were determined by the single target TaqMan® miRNA RT-qPCR assay. The ratios of all possible miRNA pairs were calculated (2^-ΔCq^) and their ability to differentiate patients with a pulmonary pathology from controls was analyzed. Figure 2, Table 1, and Additional file 1: Figure S2 demonstrate that certain miRNA pairs effectively differentiate patients with the pulmonary system pathologies from controls – both when all pathologies are grouped together, and when analyzed separately. These effective pairs include pulmonary system-enriched miRNAs paired with ubiquitous *miR-409-3p*, and pulmonary system-enriched miRNAs paired with each other.

Unexpectedly, *miR-192*, which is not enriched in the pulmonary system, paired with various miRNAs also distinguished patients with pulmonary pathologies from controls (see Additional file 1: Figure S2, panels C-F and K-L). This effect is most likely caused by *miR-192* overexpression in lung cancer and possibly other pulmonary pathologies [30,31].

The ROC curves for selected miRNA pairs and their combinations are presented in Figure 2, panel F and Additional file 1: Figure S2, panels J and L. The areas under the ROC curves (AUC) for *miR-486-5p/miR-409-3p*, *miR-486-5p/miR-146b-5p* and *miR-34b/mir-146b-5p* are 0.94-0.97, and overall accuracy is 90%-96%. P-values presented in Table 1 are 10^3^-10^7^ times lower than P < 0.0018 considered significant with Bonferroni correction (see Methods). Additional file 1: Figure S2 reports additional miRNA pairs able to effectively differentiate patients with the pulmonary system pathologies from controls.

### Differentiation between pathologies of the GI and pulmonary systems

In the next sets of experiments miRNA levels were measured in all plasma samples (40 samples of patients with pathologies of GI system, 30 samples of patients with the pulmonary system pathologies, and 30 healthy controls). In addition to miRNAs enriched in GI and pulmonary systems and several ubiquitous miRNAs, miRNAs associated with carcinogenesis and inflammation, the two types of pathologies included in the present study, were also analyzed (see Additional file 1: Table S2).

**Figure 3:**
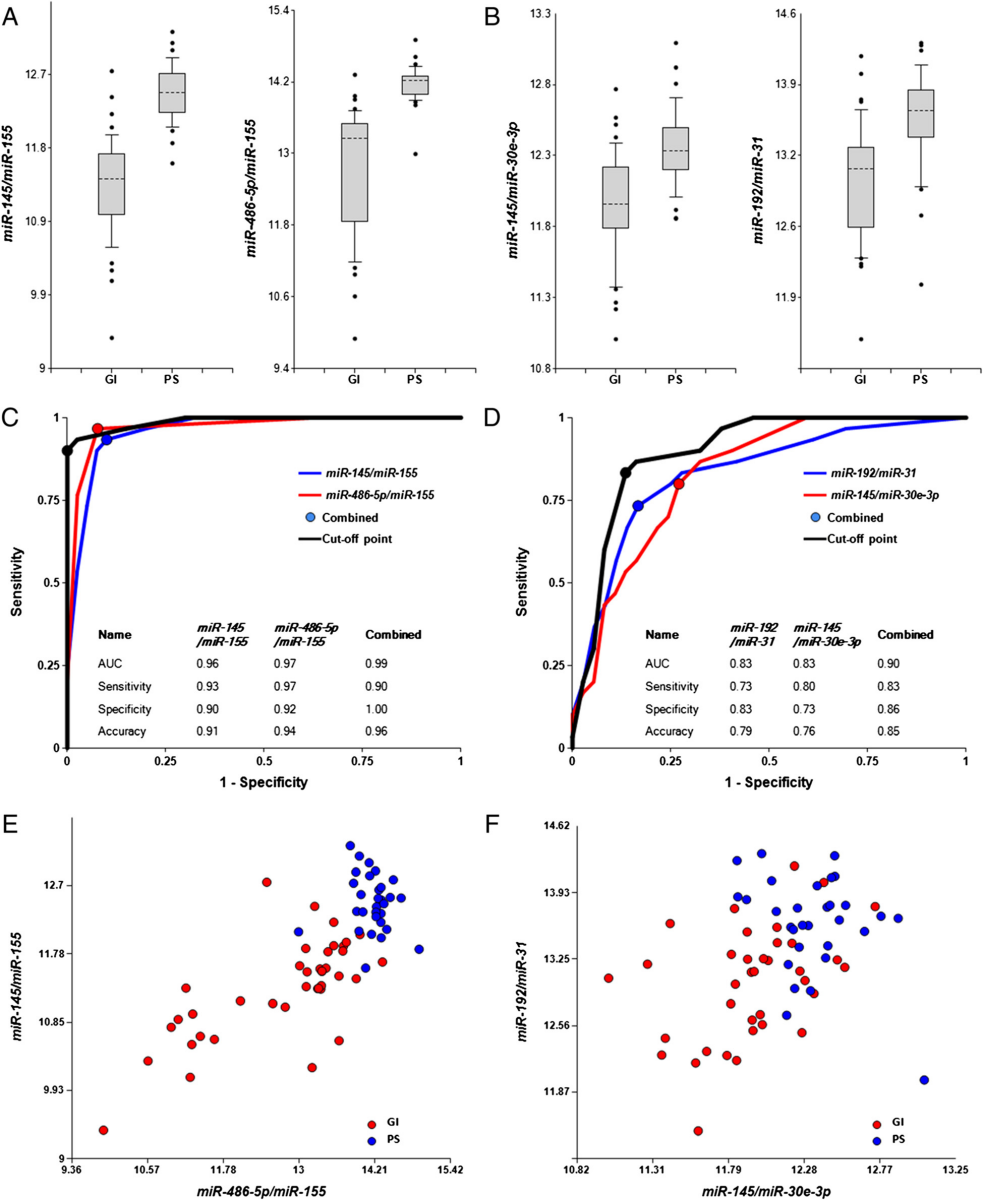
**Differentiation of GI pathologies fromPS pathologies by miRNA biomarker pairs.** The concentrations of miRNAs in plasma samples from patients with GI pathologies (40 patients total) and pulmonary system pathologies (30 patients total) were measured by RT-qPCR and the ratios of various miRNAs were calculated as 2^-ΔCq^ x100. **A**, **B** – box-plots. **C**, **D** –ROC curves for differentiation between patients with the four GI and the three pulmonary system (PS) pathologies obtained with different biomarker pairs. All statistical analyses are performed as in Figure 1. **E**, **F** – 2D-graphs comparing biomarker miRNA pairs from **A**, **C** and **B**, **D**, respectively.

Figure 3 demonstrates that the ratios of several miRNA pairs successfully differentiate pathologies of GI and pulmonary systems. The ROC curves for some miRNA pairs and their combinations are presented in Figure 3, panels C and D. The areas under the ROC curves are 0.83-0.99, and overall accuracy is 85%-96%. P-values presented in Table 1 are much lower than P = 0.0003 considered significant with Bonferroni correction (see Methods).

We have also tested the ability of selected miRNA pairs to differentiate pathologies of organs of different organ systems based on comparison of samples with a particular disease of one organ with all pathologies of another organ system grouped together. Additional file 1: Figure S3 represents respective ROC curves. The data demonstrate high efficiency of this approach, further improved by combinations of several biomarker pairs. AUC and overall accuracy are in the ranges of 0.97-1.0 and 93%-100%, respectively.

### Differentiation between cancer- and inflammation-associated pathologies

**Figure 4:**
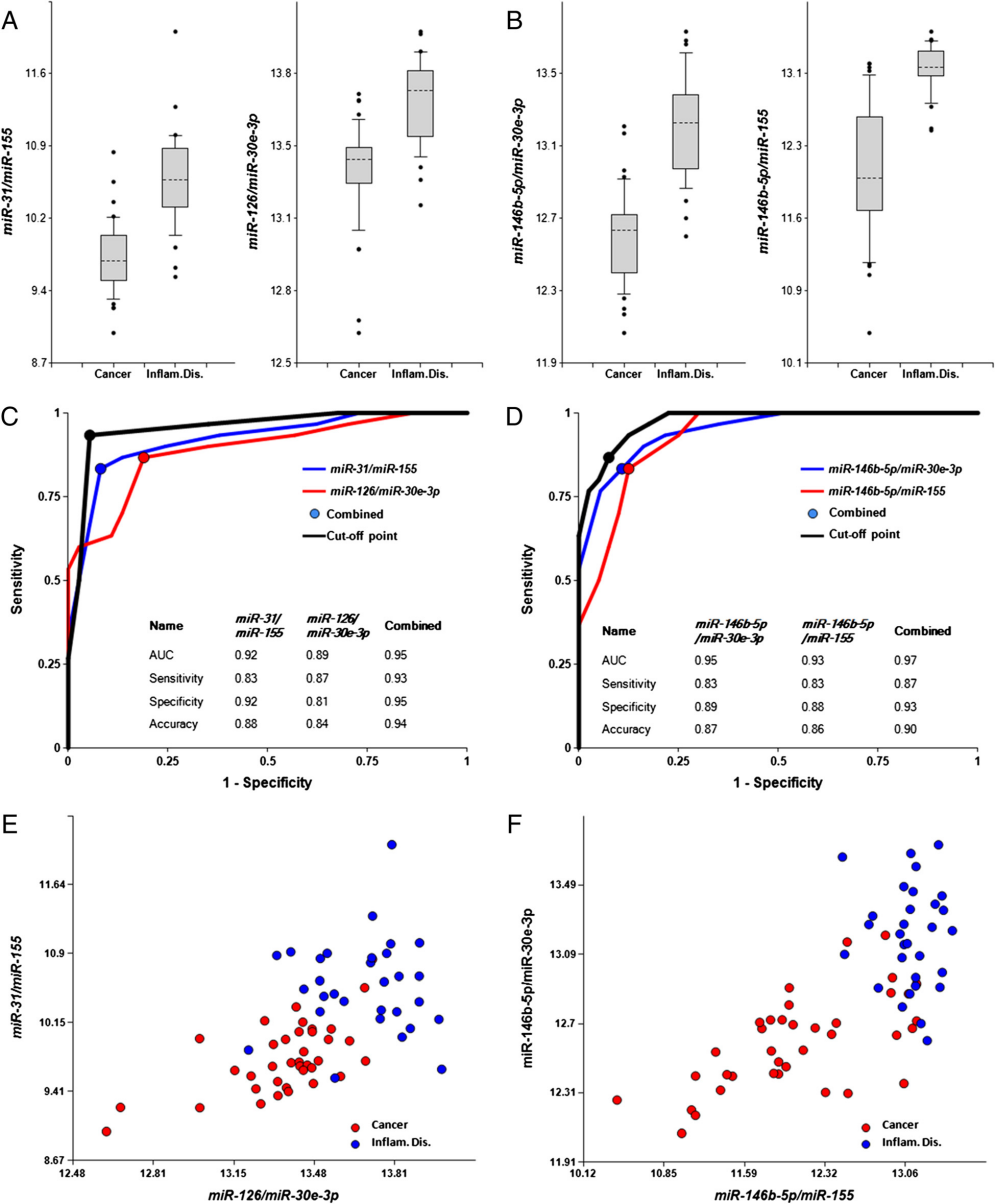
**Differentiation of all cancers from all inflammatory pathologies by miRNA biomarker pairs.** The concentrations of miRNAs in plasma samples from patients with cancers of four organs (40 patients total) and three inflammatory diseases (30 patients total) were measured by RT-qPCR and the ratios of various miRNAs were calculated as 2^-ΔCq^ x 100. **A**, **B** – box-plots. **C**, **D** – Receiver-Operating Characteristic (ROC) curves of differentiation between patients with cancers of the four organs and patients with any of the three inflammatory diseases obtained with different biomarker pairs. All statistical analyses are performed as in Figure 1. **E**, **F** – 2D-graphs comparing biomarker miRNA pairs from **A**, **C** and **B**, **D**, respectively.

Figure 4 and Table 1 reports miRNA pairs differentiating inflammation-associated pathologies (asthma, pneumonia and Crohn’s disease) grouped together from cancer (NSCLC, esophageal, gastric and colon cancers) with 87%-93% sensitivity, 93%-95% specificity, and 90%-94% overall accuracy. P-values presented in Table 1 are 10^4^-10^6^ times lower than P = 0.0003 considered significant with Bonferroni correction (see Methods). The high values obtained here for sensitivity, specificity, and accuracy are particularly notable since cancer can be accompanied by inflammation. The differentiation between two types of pathologies is even stronger when analyzed for GI and pulmonary systems separately (Additional file 1: Figure S4): AUC is 0.99 and 1.0 for pulmonary and GI systems, respectively.

## Discussion

The goal of medical screening is the detection of a disease in subjects at asymptomatic stages, which leads to more effective treatment of the disease. Two major considerations limiting application of screening tests are economical and psychological in nature. From economical perspective, the factors limiting the utility of screening tests are: (i) cost, especially when large populations are screened for rare diseases; and (ii) practically unavoidable false positive results with additional expenses for subsequent diagnostic investigations. The latter issue also leads to negative psychological effects, such as unwarranted stress and anxiety.

To address these issues, we propose a novel approach to screening, namely Universal Screening Test (UST), to detect pathologic processes in organ systems, organs and tissues. Such a test will not diagnose specific diseases, but rather will detect the presence of a pathology in a particular organ or organ system. Additional diagnostic tests will then be applied to a limited number of subjects pre-selected by UST. The present paper describes an initial study demonstrating feasibility of detection of various pathologies of a particular organ system by analysis of levels of circulating cell-free miRNAs in plasma.

There have been numerous attempts to develop screening and diagnostic tests based on analysis of circulating miRNAs. In many cases samples from patients with a particular disease were successfully differentiated from controls [5,19,24,30]. However, such findings by themselves do not necessarily mean that the tests specifically detect the disease. Many miRNAs are associated with a common pathology type, e.g. cancer, inflammation, or hypoxia and the same circulating miRNAs have been described as potential biomarkers of different diseases. For example, changes of *miR-155* concentrations were found in the bloodstream of patients with breast, esophageal, lung, pancreatic cancers and lymphomas [25,27]. Level of *miR-21* increases in plasma/serum of patients with osteosarcoma, bladder, esophageal, gastric, lung, breast, colorectal cancers, neck squamous cell carcinoma and other tumors [25-27]. Some miRNAs are associated with both cancer and inflammation [29,32-35]. A large number of similar examples can be found in literature. In summary, a mere differentiation of patients with a disease from control subjects is not sufficient for specific detection of a disease in a clinical setting. Additional information, such as localization of a disease in a particular organ, is required.

Here we proposed to analyze organ/organ system-enriched miRNAs to differentiate pathological processes of different organs, based on a hypothesis that changes in their concentrations in plasma will most likely be associated with a disease of a respective organ or organ system. We also included in the study a number of miRNAs involved in such broad classes of pathologies as carcinogenesis and inflammation, to find out whether analysis of the levels of these miRNAs along with the levels of organ-enriched miRNAs can provide more specific information about pathology.

In the current study analysis of miRNA ratios (miRNA biomarker pairs) was performed to account for numerous factors that affect detectable levels of various miRNAs: miRNA stability in plasma, effectiveness of its purification, potential RT-qPCR inhibition, etc. Further, calculation of ratios of two miRNAs could increase sensitivity and specificity of a biomarker panel, since expression and secretion of different miRNAs may be affected differently (e.g. changed in opposite directions) by pathology.

Two organ systems and seven pathologies were used to test the proposed approach. The results obtained can be summarized as follows: (1) analysis of organ system-enriched miRNA levels in plasma effectively detected patients with the pathologies studied: asthma, pneumonia and NSCLC in pulmonary system and Crohn’s disease, esophageal, gastric and colon cancers in GI system; it is important to note that all 30 cancers of the GI system and 4 out of 10 NSCLC were in stages I/II (see Additional file 1: Table S1), providing a dataset with relatively early stages of pathology; (2) miRNA pairs successfully differentiating patients with pulmonary system diseases from patients with the GI system diseases have been identified; and (3) cancer patients were effectively distinguished from patients with inflammatory diseases.

We have recently demonstrated that a similar approach can be used for early detection of MCI, a syndrome characteristic of early stages of various neurodegenerative diseases, including Alzheimer’s and Parkinson’s diseases [14]. Two sets of biomarker miRNA pairs capable of differentiating MCI from age matched controls with 82%-92% accuracy were found. These biomarkers were then successfully validated in a larger study with independent cohorts of plasma samples, and accuracy obtained in this validation study was 87% - 96% (poster presented in 2013 Alzheimer's Association International Conference; Aging, manuscript in press). In addition, in a small retrospective longitudinal study we analyzed plasma samples collected from the same patients at different time points for up to 5 years and demonstrated that MCI can be detected 1–5 years prior to clinical diagnosis [14]. The values of miRNA biomarker pairs in plasma samples collected from same subjects over the course of 1 to 5 years were very consistent.

The most common approach to search for circulating miRNA biomarkers of various diseases is miRNA array analysis of numerous miRNAs in serum or plasma. However, the concentrations of many organ-enriched miRNAs in bodily fluids are too low to be detected by miRNA arrays and, as a result, they can be missed in such studies. Nonetheless, some of these miRNAs are detectable and, in fact, these miRNAs are among the most promising biomarkers due to increase of their levels in plasma or serum. Circulating liver-enriched *miR-122*[36] and heart-enriched *miR-1*, *miR-133*, *miR-208* and *miR-499*[37] represent good examples since their concentrations have been found to be significantly higher in the bloodstream of patients with various diseases of respective organs. Interestingly, in some instances concentrations of these miRNAs in organ tissues are decreased due to pathology but their levels in plasma or serum go up [38], which additionally supports the idea of using organ-enriched miRNAs for detecting pathology of respective organs.

Thus, the data obtained in the present study and the results of literature analysis indicate that the idea of Universal Screening Test presented herein is viable. Naturally, larger studies involving patients with diseases of GI and pulmonary systems, as well as accurately matched control subjects, including cohorts with different age, gender, and ethnicity, are necessary for further validation of the approach. Longitudinal studies and analysis of precancerous conditions, such as adenomatous polyps, will help clarify how early various organ pathologies can be detected. It also would be important to compare levels of these miRNA biomarkers in subjects before, during and after treatment in order to evaluate their applicability to disease and treatment monitoring. Further, studies with additional miRNAs and patients with pathologies of other organs should be performed.

Currently, data on miRNA expression in various organs, tissues and cell types are still limited. Some miRNAs can be enriched in several organs. Further, plasma levels of miRNAs not enriched in a certain organ may increase due to higher expression or secretion caused by pathology. The increase in concentration of circulating *miR-192* in patients with pathologies of pulmonary system reported here (see Additional file 1: Figure S2) provides a good example of such an increase caused by pathology. We believe that a potential ambiguity with respect to organs or organ systems affected by pathology, as inferred from analysis of certain miRNAs, such as *miR-192*, can be overcome by measuring levels of several miRNAs enriched in each organ system, organ and tissue.

**Figure 5:**
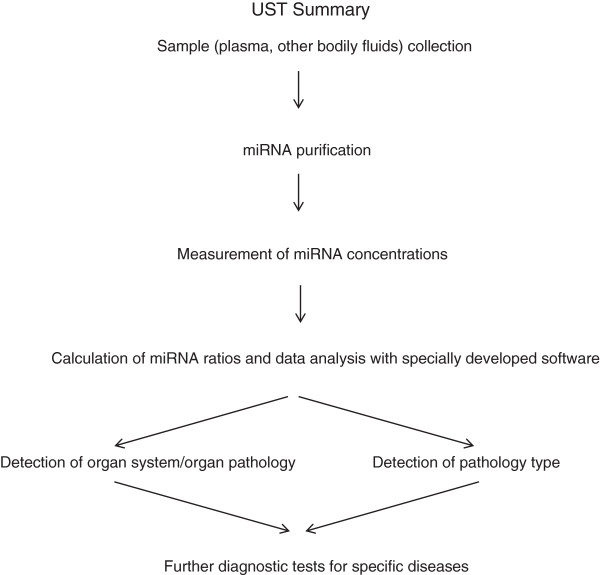
The UST workflow.

Figure 5 presents a diagram of the proposed UST workflow. Briefly, after measuring miRNA concentrations in a bodily fluid, pathologies in one or several organ systems are detected based on calculations of miRNA ratios. When possible, a pathology type (e.g. cancer, inflammation) is determined. If pathology is detected, specific diagnostic tests should be performed.

## Conclusions

The present paper describes an approach to the development of Universal Screening Test for early detection of pathological processes in a particular organ or organ system based on analysis of circulating organ-enriched miRNAs, and, as a feasibility study, reports identification of plasma miRNA biomarkers for detection of diseases of gastrointestinal and pulmonary systems.

## Abbreviations

(UST): Universal screening test; (GI): Gastrointestinal; (PS): Pulmonary system; (RT-qPCR): Reverse transcription quantitative polymerase chain reaction; (ROC): Receiver operating characteristic; (AUC): Area under the receiver operating characteristic curve.

## Competing interests

All authors are shareholders of DiamiR and co-inventors on a patent application related to the manuscript subject: Sheinerman Kira S., Tsivinsky Vladimir G., Umansky Samuil R., miRNA-based Universal Screening Test (UST). International Publication Number: WO 2012/145409 A1. International Filing Date: April 18, 2012.

## Authors’ contributions

Conceived and designed the experiments, analyzed the data: K.S.S. and S.R.U. Developed the software and performed bioinformatics analysis: V.G.T. Wrote the paper: K.S.S. and S.R.U. All authors read and approved the final manuscript.

## Supplementary Material

Additional file 1: Table S1 Demographics of plasma donors. **Table S2.** miRNAs used in the study. **Figure S1.** Differentiation of GI pathologies from controls by miRNA biomarker pairs. **Figure S2.** Differentiation of PS pathologies from controls by miRNA biomarker pairs. **Figure S3.** ROC curve analysis for differentiation between particular diseases of one organ system and combined pathologies of another organ system, obtained with different biomarker pairs. **Figure S4.** ROC curve analysis for differentiation of cancer(s) from inflammatory disease(s) of GI (A) and PS (B), obtained with different biomarker pairs. Click here for file
