# Supplementary material for: Analysis of organ-enriched microRNAs in plasma as an approach to development of Universal Screening Test: feasibility study
Source: J Transl Med. 2013 Dec 11;11:304. doi: 10.1186/1479-5876-11-304 (PMC3867418; doi:10.1186/1479-5876-11-304)
Supplement: Additional file 1: Table S1 — Demographics of plasma donors. Table S2. miRNAs used in the study. Figure S1. Differentiation of GI pathologies from controls by miRNA biomarker pairs. Figure S2. Differentiation of PS pathologies from controls by miRNA biomarker pairs. Figure S3. ROC curve analysis for differentiation between particular diseases of one organ system and combined pathologies of another organ system, obtained with different biomarker pairs. Figure S4. ROC curve analysis for differentiation of cancer(s) from inflammatory disease(s) of GI (A) and PS (B), obtained with different biomarker pairs. [file 1479-5876-11-304-S1.pdf]

Additional Files

Table S1. Demographics of plasma donors

Organ system	Clinical diagnosis	Number	Cancer Stage (# of Patients)	Sex Male /Female	Age Average±SD
Gastrointestinal	Esophageal cancer (EC)	10	1(2), 2(8)	8/2	56.8±2.5
	Gastric cancer (GC)	10	1(2), 2(8)	1/9	55.8±3.3
	Colon cancer (CC)	10	1(3), 2(7)	8/2	57.8±2.7
	Crohn's disease (CD)	10	NA	5/5	53.3±9.4
Pulmonary	Asthma	10	NA	0/10	61.4±12.0
	Pneumonia	10	NA	6/4	51.9±7.8
	Non-small cell lung cancer (NSCLC)	10	1(2), 2(2), 3(3), 4(3)	3/7	58.5±3.6
	Controls	30	NA	26/4	63.7±4.0

NA – Not Applicable; SD – Standard Deviation

Table S2. miRNA used in the study

miRNA	Enriched in GI	Enriched in lung	Involved in cancer	Involved in inflammation	Under-expressed in	AB Catalog Assay ID
miR-17-5p			+			002308
miR-30e-3p	Ubiquitous					000422
miR-31			+	+		002279
miR-34b		+			GI	002102
miR-126			+	+		002228
miR-142-5p		+				002248
miR-145	+					002278
miR-146b-5p		+	+	?		001097
miR-148a	+		+			000470
miR-155		+	+	+		002623
miR-181a	Ubiquitous, brain-enriched					000480
miR-192	+					000491
miR-194	+					000493
miR-203	+					000507
miR-215	+				Lung	000518
miR-223		+	?	+		002295
miR-409-3p	Ubiquitous				Lung	002332
miR-486-5p		+				001278

Figure S1

Differentiation of GI pathologies from controls by miRNA biomarker pairs.

The concentrations of miRNAs in plasma samples from patients with four GI pathologies, and from healthy donors were measured by RT-qPCR and the ratios of various miRNAs were calculated as $2^{-\Delta Cq}$ x 100. A-G – box-plots; A-F – individual pathologies (10 patients in each group) against controls (30 subjects); G – combined GI pathologies (40 patients total) against controls (30 subjects). All notations are as in Fig.1. H – Receiver-Operating Characteristic (ROC) curve for differentiation between patients with four GI pathologies and controls obtained with different biomarker pairs. All statistical analyses are performed as in Fig.1.

Figure S2

Differentiation of PS pathologies from controls by miRNA biomarker pairs.

The concentrations of miRNAs in plasma samples from patients with three PS pathologies, and from healthy donors were measured by RT-qPCR and the ratios of various miRNAs were calculated as $2^{-\Delta Cq}$ x100. **A-H**, **I** and **K** – box-plots; **A-H** – individual pathologies (10 patients in each group) against controls (20 subjects); **I** and **K** – combined PS pathologies (30 patients total) against controls (20 subjects). **J** and **L** – Receiver-Operating Characteristic (ROC) curves of differentiation between patients with three PS pathologies and controls obtained with different biomarker pairs. All statistical analyses are performed as in Fig.1.

Figure S3

Receiver-Operating Characteristic (ROC) curve analysis for differentiation between particular diseases of one organ system and combined pathologies of another organ system, obtained with different biomarker pairs.

The areas under the ROC curve (AUC) are reported. The statistical analysis is performed as in Fig.1.

Figure S4

Receiver-Operating Characteristic (ROC) curve analysis for differentiation of cancer(s) from inflammatory disease(s) of GI (A) and PS (B), obtained with different biomarker pairs.

A – Patients with Crohn’s disease versus patients with cancers of the GI system; B – Patients with lung cancer versus patients with either asthma or pneumonia. The areas under the ROC curve (AUC) are reported. The statistical analysis is performed as in Fig.1.

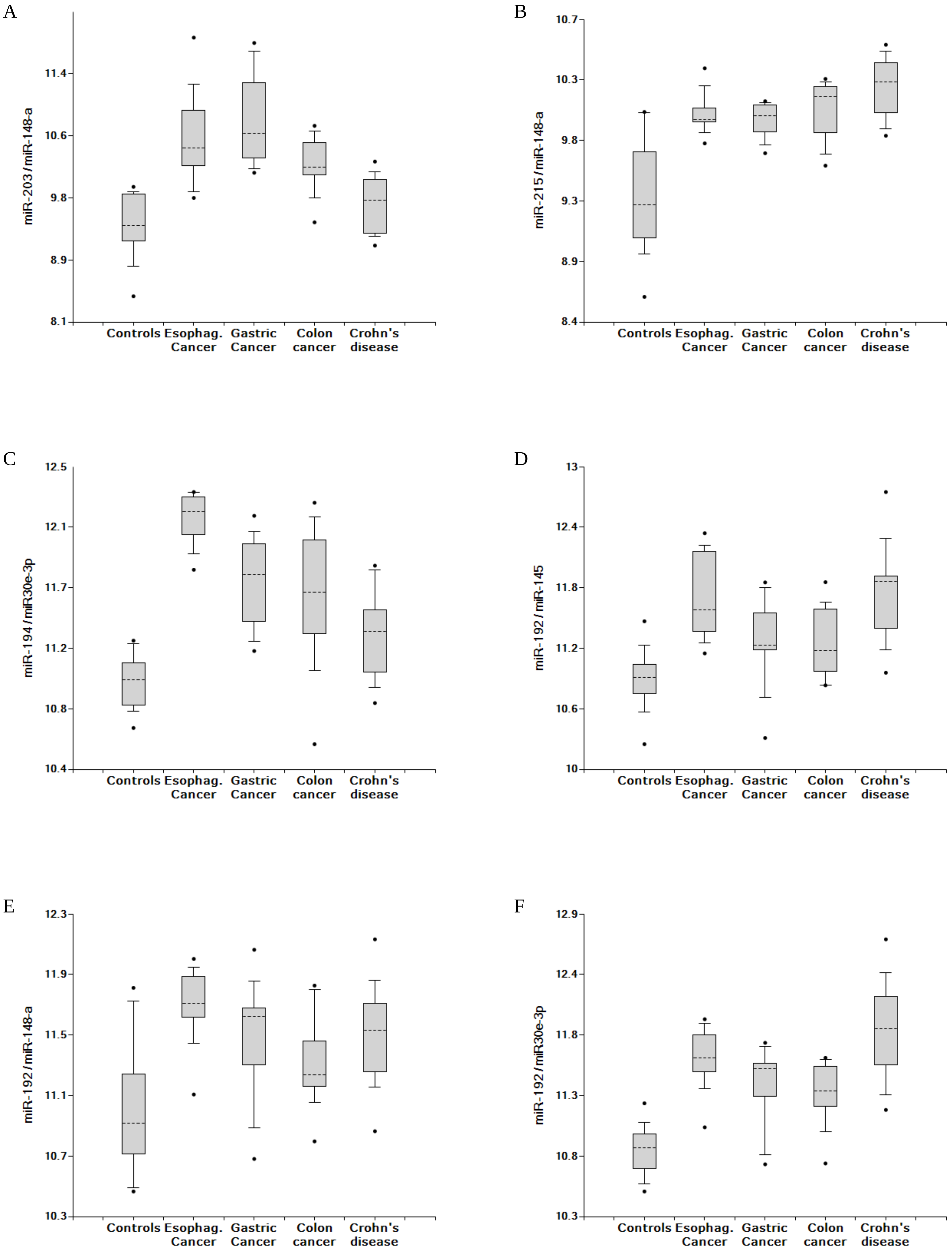

A
miR-203 / miR-148-a
11.4
10.6
9.8
8.9
8.1
Controls
Esophag. Cancer
Gastric Cancer
Colon cancer
Crohn's disease
B
miR-215 / miR-148-a
10.7
10.3
9.8
9.3
8.9
8.4
Controls
Esophag. Cancer
Gastric Cancer
Colon cancer
Crohn's disease
C
miR-194 / miR30e-3p
12.5
12.1
11.7
11.2
10.8
10.4
Controls
Esophag. Cancer
Gastric Cancer
Colon cancer
Crohn's disease
D
miR-192 / miR-145
13
12.4
11.8
11.2
10.6
10
Controls
Esophag. Cancer
Gastric Cancer
Colon cancer
Crohn's disease
E
miR-192 / miR-148-a
12.3
11.9
11.5
11.1
10.7
10.3
Controls
Esophag. Cancer
Gastric Cancer
Colon cancer
Crohn's disease
F
miR-192 / miR30e-3p
12.9
12.4
11.8
11.3
10.8
10.3
Controls
Esophag. Cancer
Gastric Cancer
Colon cancer
Crohn's disease

G

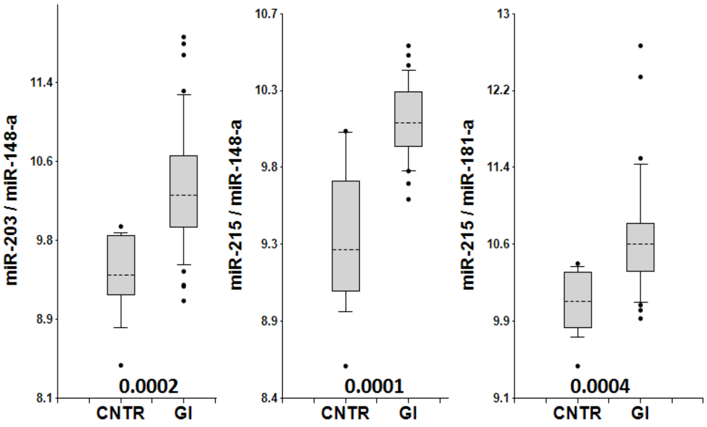

miR-203 / miR-148-a
miR-215 / miR-148-a
miR-215 / miR-181-a
11.4
10.6
9.8
8.9
8.1
10.7
10.3
9.8
9.3
8.9
8.4
13
12.2
11.4
10.6
9.9
9.1
0.0002
0.0001
0.0004
CNTR
GI

H

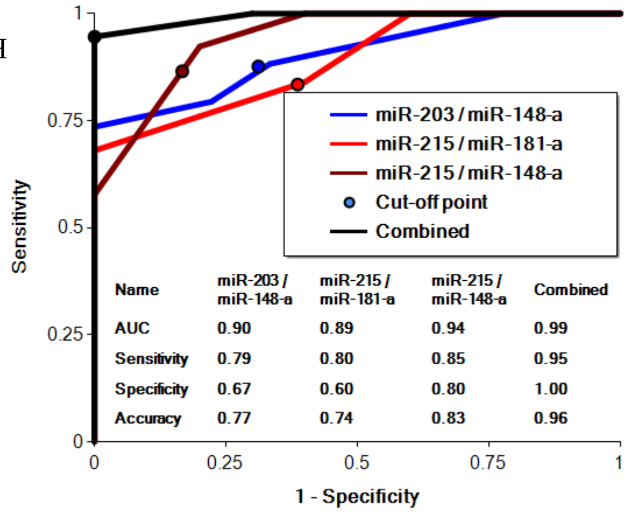

miR-203 / miR-148-a
miR-215 / miR-181-a
miR-215 / miR-148-a
Cut-off point
Combined
Sensitivity
1 - Specificity
0
0.25
0.5
0.75
1
Name | miR-203 / miR-148-a | miR-215 / miR-181-a | miR-215 / miR-148-a | Combined
AUC | 0.90 | 0.89 | 0.94 | 0.99
Sensitivity | 0.79 | 0.80 | 0.85 | 0.95
Specificity | 0.67 | 0.60 | 0.80 | 1.00
Accuracy | 0.77 | 0.74 | 0.83 | 0.96

Figure S1

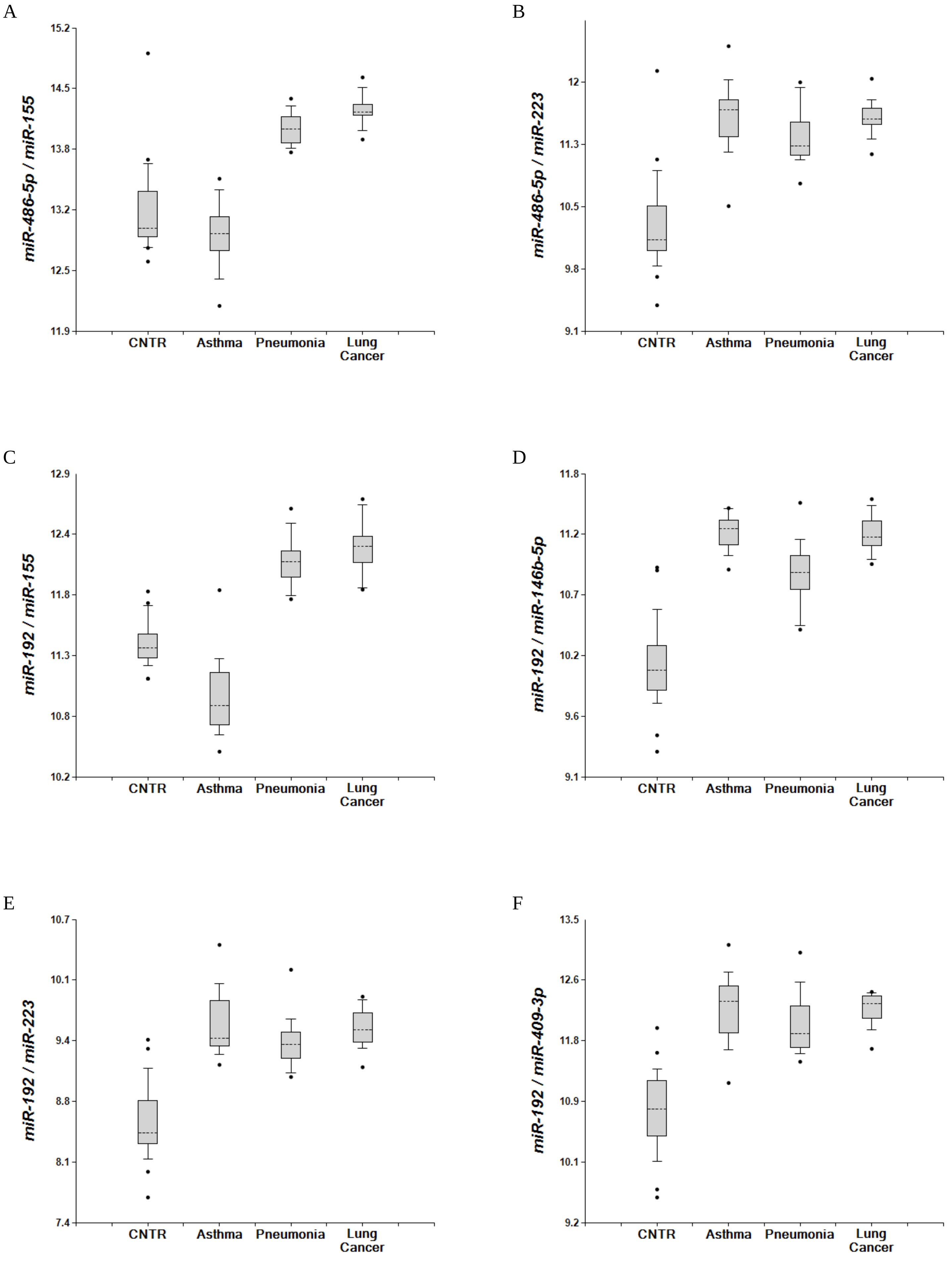
A
miR-486-5p / miR-155
15.2
14.5
13.8
13.2
12.5
11.9
B
miR-486-5p / miR-223
12
11.3
10.5
9.8
9.1
C
miR-192 / miR-155
12.9
12.4
11.8
11.3
10.8
10.2
D
miR-192 / miR-146b-5p
11.8
11.2
10.7
10.2
9.6
9.1
E
miR-192 / miR-223
10.7
10.1
9.4
8.8
8.1
7.4
F
miR-192 / miR-409-3p
13.5
12.6
11.8
10.9
10.1
9.2
CNTR
Asthma
Pneumonia
Lung Cancer

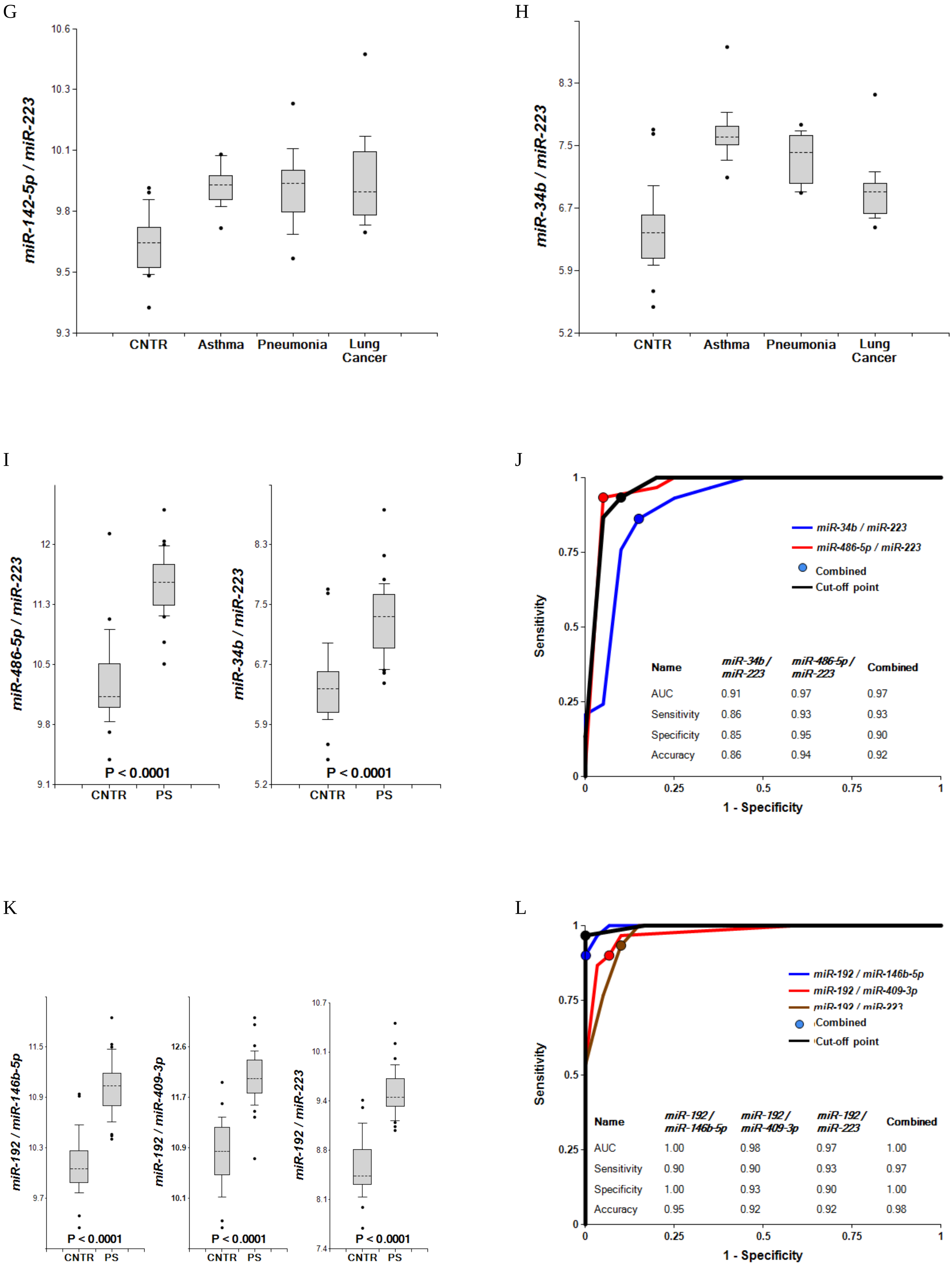
G
miR-142-5p / miR-223
10.6
10.3
10.1
9.8
9.5
9.3
CNTR
Asthma
Pneumonia
Lung Cancer
H
miR-34b / miR-223
8.3
7.5
6.7
5.9
5.2
CNTR
Asthma
Pneumonia
Lung Cancer
I
miR-486-5p / miR-223
12
11.3
10.5
9.8
9.1
P < 0.0001
CNTR
PS
miR-34b / miR-223
8.3
7.5
6.7
5.9
5.2
P < 0.0001
CNTR
PS
J
Sensitivity
1 - Specificity
miR-34b / miR-223
miR-486-5p / miR-223
Combined
Cut-off point
Name | miR-34b / miR-223 | miR-486-5p / miR-223 | Combined
AUC | 0.91 | 0.97 | 0.97
Sensitivity | 0.86 | 0.93 | 0.93
Specificity | 0.85 | 0.95 | 0.90
Accuracy | 0.86 | 0.94 | 0.92
K
miR-192 / miR-146b-5p
11.5
10.9
10.3
9.7
P < 0.0001
CNTR
PS
miR-192 / miR-409-3p
12.6
11.7
10.9
10.1
P < 0.0001
CNTR
PS
miR-192 / miR-223
10.7
10.1
9.4
8.8
8.1
7.4
P < 0.0001
CNTR
PS
L
Sensitivity
1 - Specificity
miR-192 / miR-146b-5p
miR-192 / miR-409-3p
miR-192 / miR-223
Combined
Cut-off point
Name | miR-192 / miR-146b-5p | miR-192 / miR-409-3p | miR-192 / miR-223 | Combined
AUC | 1.00 | 0.98 | 0.97 | 1.00
Sensitivity | 0.90 | 0.90 | 0.93 | 0.97
Specificity | 1.00 | 0.93 | 0.90 | 1.00
Accuracy | 0.95 | 0.92 | 0.92 | 0.98

Figure S2

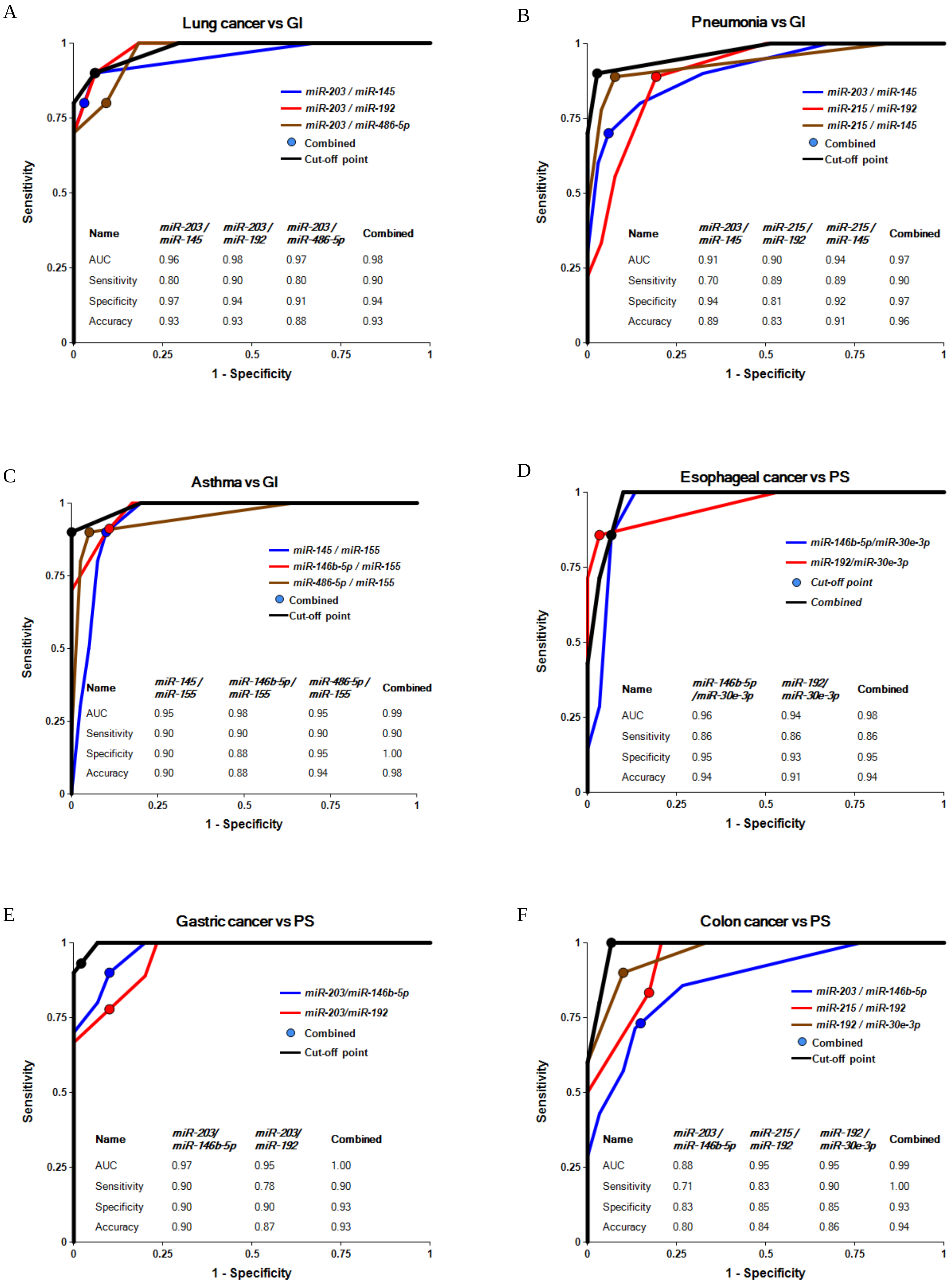
A
Lung cancer vs GI
miR-203 / miR-145
miR-203 / miR-192
miR-203 / miR-486-5p
Combined
Cut-off point
Sensitivity
1 - Specificity
Name miR-203 / miR-145 miR-203 / miR-192 miR-203 / miR-486-5p Combined
AUC 0.96 0.98 0.97 0.98
Sensitivity 0.80 0.90 0.80 0.90
Specificity 0.97 0.94 0.91 0.94
Accuracy 0.93 0.93 0.88 0.93
B
Pneumonia vs GI
miR-203 / miR-145
miR-215 / miR-192
miR-215 / miR-145
Combined
Cut-off point
Sensitivity
1 - Specificity
Name miR-203 / miR-145 miR-215 / miR-192 miR-215 / miR-145 Combined
AUC 0.91 0.90 0.94 0.97
Sensitivity 0.70 0.89 0.89 0.90
Specificity 0.94 0.81 0.92 0.97
Accuracy 0.89 0.83 0.91 0.96
C
Asthma vs GI
miR-145 / miR-155
miR-146b-5p / miR-155
miR-486-5p / miR-155
Combined
Cut-off point
Sensitivity
1 - Specificity
Name miR-145 / miR-155 miR-146b-5p / miR-155 miR-486-5p / miR-155 Combined
AUC 0.95 0.98 0.95 0.99
Sensitivity 0.90 0.90 0.90 0.90
Specificity 0.90 0.88 0.95 1.00
Accuracy 0.90 0.88 0.94 0.98
D
Esophageal cancer vs PS
miR-146b-5p/miR-30e-3p
miR-192/miR-30e-3p
Cut-off point
Combined
Sensitivity
1 - Specificity
Name miR-146b-5p /miR-30e-3p miR-192/ miR-30e-3p Combined
AUC 0.96 0.94 0.98
Sensitivity 0.86 0.86 0.86
Specificity 0.95 0.93 0.95
Accuracy 0.94 0.91 0.94
E
Gastric cancer vs PS
miR-203/miR-146b-5p
miR-203/miR-192
Combined
Cut-off point
Sensitivity
1 - Specificity
Name miR-203/ miR-146b-5p miR-203/ miR-192 Combined
AUC 0.97 0.95 1.00
Sensitivity 0.90 0.78 0.90
Specificity 0.90 0.90 0.93
Accuracy 0.90 0.87 0.93
F
Colon cancer vs PS
miR-203 / miR-146b-5p
miR-215 / miR-192
miR-192 / miR-30e-3p
Combined
Cut-off point
Sensitivity
1 - Specificity
Name miR-203 / miR-146b-5p miR-215 / miR-192 miR-192 / miR-30e-3p Combined
AUC 0.88 0.95 0.95 0.99
Sensitivity 0.71 0.83 0.90 1.00
Specificity 0.83 0.85 0.85 0.93
Accuracy 0.80 0.84 0.86 0.94

G

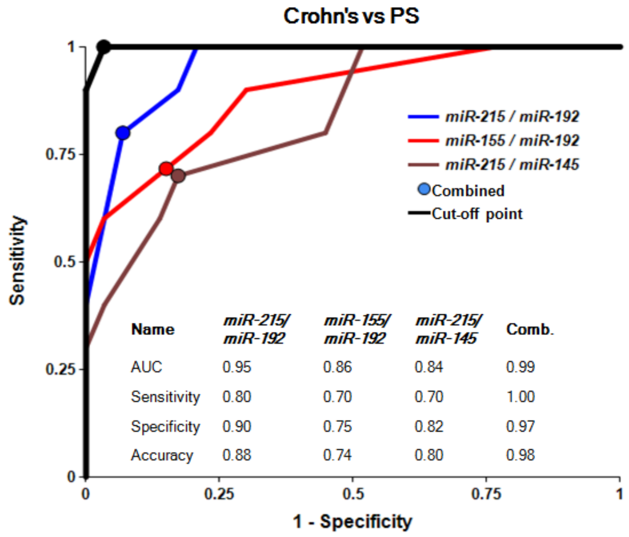
Crohn's vs PS
Sensitivity
1 - Specificity
miR-215 / miR-192
miR-155 / miR-192
miR-215 / miR-145
Combined
Cut-off point
Name
miR-215/ miR-192
miR-155/ miR-192
miR-215/ miR-145
Comb.
AUC 0.95 0.86 0.84 0.99
Sensitivity 0.80 0.70 0.70 1.00
Specificity 0.90 0.75 0.82 0.97
Accuracy 0.88 0.74 0.80 0.98

Figure S3

A

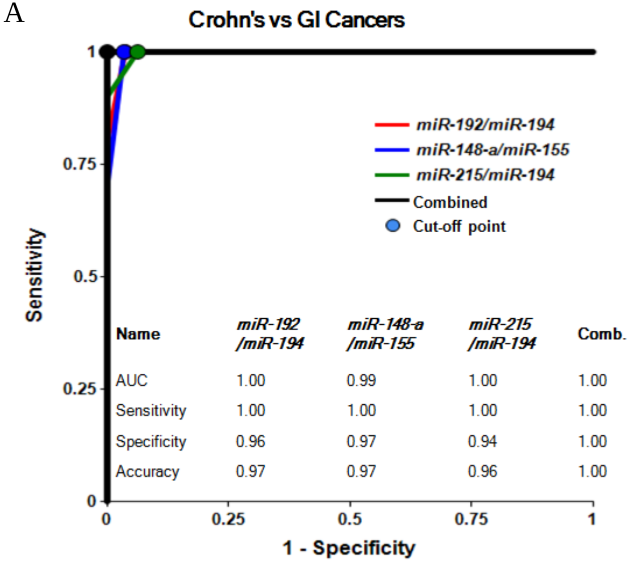
Crohn's vs GI Cancers
miR-192/miR-194
miR-148-a/miR-155
miR-215/miR-194
Combined
Cut-off point
Sensitivity
1 - Specificity
Name | miR-192 /miR-194 | miR-148-a /miR-155 | miR-215 /miR-194 | Comb.
AUC | 1.00 | 0.99 | 1.00 | 1.00
Sensitivity | 1.00 | 1.00 | 1.00 | 1.00
Specificity | 0.96 | 0.97 | 0.94 | 1.00
Accuracy | 0.97 | 0.97 | 0.96 | 1.00

B

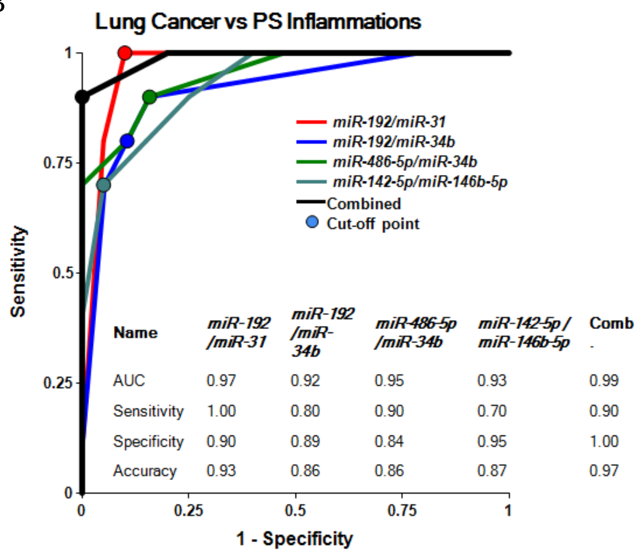
Lung Cancer vs PS Inflammations
miR-192/miR-31
miR-192/miR-34b
miR-486-5p/miR-34b
miR-142-5p/miR-146b-5p
Combined
Cut-off point
Sensitivity
1 - Specificity
Name | miR-192 /miR-31 | miR-192 /miR-34b | miR-486-5p /miR-34b | miR-142-5p / miR-146b-5p | Comb.
AUC | 0.97 | 0.92 | 0.95 | 0.93 | 0.99
Sensitivity | 1.00 | 0.80 | 0.90 | 0.70 | 0.90
Specificity | 0.90 | 0.89 | 0.84 | 0.95 | 1.00
Accuracy | 0.93 | 0.86 | 0.86 | 0.87 | 0.97

Figure S4
